# Malnutrition and Poor Physical Function Are Associated With Higher Comorbidity Index in Hospitalized Older Adults

**DOI:** 10.3389/fnut.2022.920485

**Published:** 2022-06-24

**Authors:** Maria Amasene, María Medrano, Iñaki Echeverria, Miriam Urquiza, Ana Rodriguez-Larrad, Amaia Diez, Idoia Labayen, Besga-Basterra Ariadna

**Affiliations:** ^1^Department of Pharmacy and Food Science, University of the Basque Country UPV/EHU, Vitoria-Gasteiz, Spain; ^2^Institute on Innovation and Sustainable Development in Food Chain (IS-FOOD), Public University of Navarre (UPNA), Pamplona, Spain; ^3^Department of Physiology, University of the Basque Country, UPV/EHU, Leioa, Spain; ^4^Department of Physical Education and Sport, University of the Basque Country, Vitoria-Gasteiz, Spain; ^5^Biocruces Bizkaia Health Research Institute, Barakaldo, Spain; ^6^Nurse Supervisor, Bioaraba Research Institute, Araba University Hospital, Vitoria-Gasteiz, Spain; ^7^Ageing and Frailty Research Group, Bioaraba Health Research Institute, Vitoria-Gasteiz, Spain; ^8^Internal Medicine Department, Osakidetza Basque Health Service, Araba University Hospital, Vitoria-Gasteiz, Spain

**Keywords:** chronic diseases, geriatrics, inpatients, nutritional status, muscle strength, mortality

## Abstract

**Background:**

The Charlson Comorbidity Index (CCI) is the most widely used method to measure comorbidity and predict mortality. There is no evidence whether malnutrition and/or poor physical function are associated with higher CCI in hospitalized patients. Therefore, this study aimed to (i) analyze the association between the CCI with nutritional status and with physical function of hospitalized older adults and (ii) examine the individual and combined associations of nutritional status and physical function of older inpatients with comorbidity risk.

**Methods:**

A total of 597 hospitalized older adults (84.3 ± 6.8 years, 50.3% women) were assessed for CCI, nutritional status (the Mini Nutritional Assessment-Short Form [MNA-SF]), and physical function (handgrip strength and the Short Physical Performance Battery [SPPB]).

**Results:**

Better nutritional status (*p* < 0.05) and performance with handgrip strength and the SPPB were significantly associated with lower CCI scores among both men (*p* < 0.005) and women (*p* < 0.001). Patients with malnutrition or risk of malnutrition (*OR*: 2.165, 95% *CI*: 1.408–3.331, *p* < 0.001) as well as frailty (*OR*: 3.918, 95% *CI*: 2.326–6.600, *p* < 0.001) had significantly increased the risk for being at severe risk of comorbidity. Patients at risk of malnutrition or that are malnourished had higher CCI scores regardless of being fit or unfit according to handgrip strength (*p* for trend < 0.05), and patients classified as frail had higher CCI despite their nutritional status (*p* for trend < 0.001).

**Conclusions:**

The current study reinforces the use of the MNA-SF and the SPPB in geriatric hospital patients as they might help to predict poor clinical outcomes and thus indirectly predict post-discharge mortality risk.

## Introduction

The prevalence of chronic diseases has substantially increased in the last years along with the aging of the population (1, 2). It has been reported that, in Europe, 50% of older adults have ≥2 chronic diseases (3).

Hospitalization rates increase linearly with the number of chronic diseases (4) and thereby healthcare costs (5). In clinical settings, the Charlson Comorbidity Index (CCI) is the most widely used method to measure comorbidity and predict mortality (6). This index considers the number and severity of the concurrent diseases with the aim of identifying those patients at risk for negative health outcomes (4).

Malnutrition is often observed among older adults at hospital admission (7), hindering recovery from diseases, surgery, or trauma, worsening the prognosis (8, 9) and increasing the healthcare costs (5). Hence, being malnourished has been associated with a higher risk of in-hospital mortality (9, 10) as well as with higher mortality in the short- (11–14) and the long-term after discharge (10, 14, 15).

Several performance-based physical tests have shown good validity for predicting poor health outcomes (16, 17). Handgrip strength has been proposed as an important biomarker of health status (18) and a potential predictor of comorbidity (19, 20) and mortality (21–23). Similarly, the Short Physical Performance Battery (SPPB) seems to be able to predict disability (24) and mortality risk (25, 26).

Thereby, although it is not new that malnutrition and markers of physical function contribute to increasing comorbidity (18, 27) and lastly mortality (25, 27, 28), there are few studies aiming to evaluate the associations of comorbidity assessed by CCI with malnutrition and poor physical function (13, 29, 30). Hence, there are no previous studies examining whether malnutrition and poor physical function, independently or in combination, are associated with higher CCI in hospitalized patients. Therefore, this study aimed to (i) analyze the association between the CCI, nutritional status, and physical function of hospitalized older adults and (ii) examine the individual and combined associations of nutritional status and physical function of older inpatients with comorbidity risk.

## Methods

### Study Design

This cross-sectional study is a secondary analysis, with the CCI variable as the endpoint of the study. This study was based on the data obtained during the recruitment for a randomized controlled trial (ClinicalTrials.gov ID: NCT03815201), which was conducted in Vitoria-Gasteiz (North of Spain) at the internal medicine service of the Araba University Hospital from September 2017 to August 2018. This study was approved by the Clinical Research Ethics Committee of the Araba University Hospital (CEIC-HUA: 2017-021) and is in line with the revised ethical guidelines of the Declaration of Helsinki (revision of 2013). All patients were informed about the details of the research and signed informed consent before participating in the study.

### Participants

The daily list of hospitalized patients at the internal medicine service was revisited to assess whether patients were eligible for evaluation or not by members of the research team with a wide experience in clinical settings. Patients were eligible for evaluation if they met the following inclusion criteria: ≥70 years old, a score of ≥20 on the Mini-Mental State Questionnaire (MMSE), were able to walk alone or using a walking stick or walking frame, and were able to understand and follow the instructions. However, they were not eligible for evaluation if they had any of the following exclusion criteria: been suffering from severe dementia or Parkinson's disease, been unable to stand and/or walk a short distance, been in a critical medical condition (e.g., a need of palliative care and/or advanced cancer) or death, and suffered any fracture of the upper or lower limbs in the last 3 months. Hence, patients with no valid data regarding nutritional status [assessed by the Mini Nutritional Assessment-Short Form (MNA-SF)], CCI, and physical function (neither data for handgrip strength nor SPPB) were excluded for analysis in this study.

**Figure 1:**
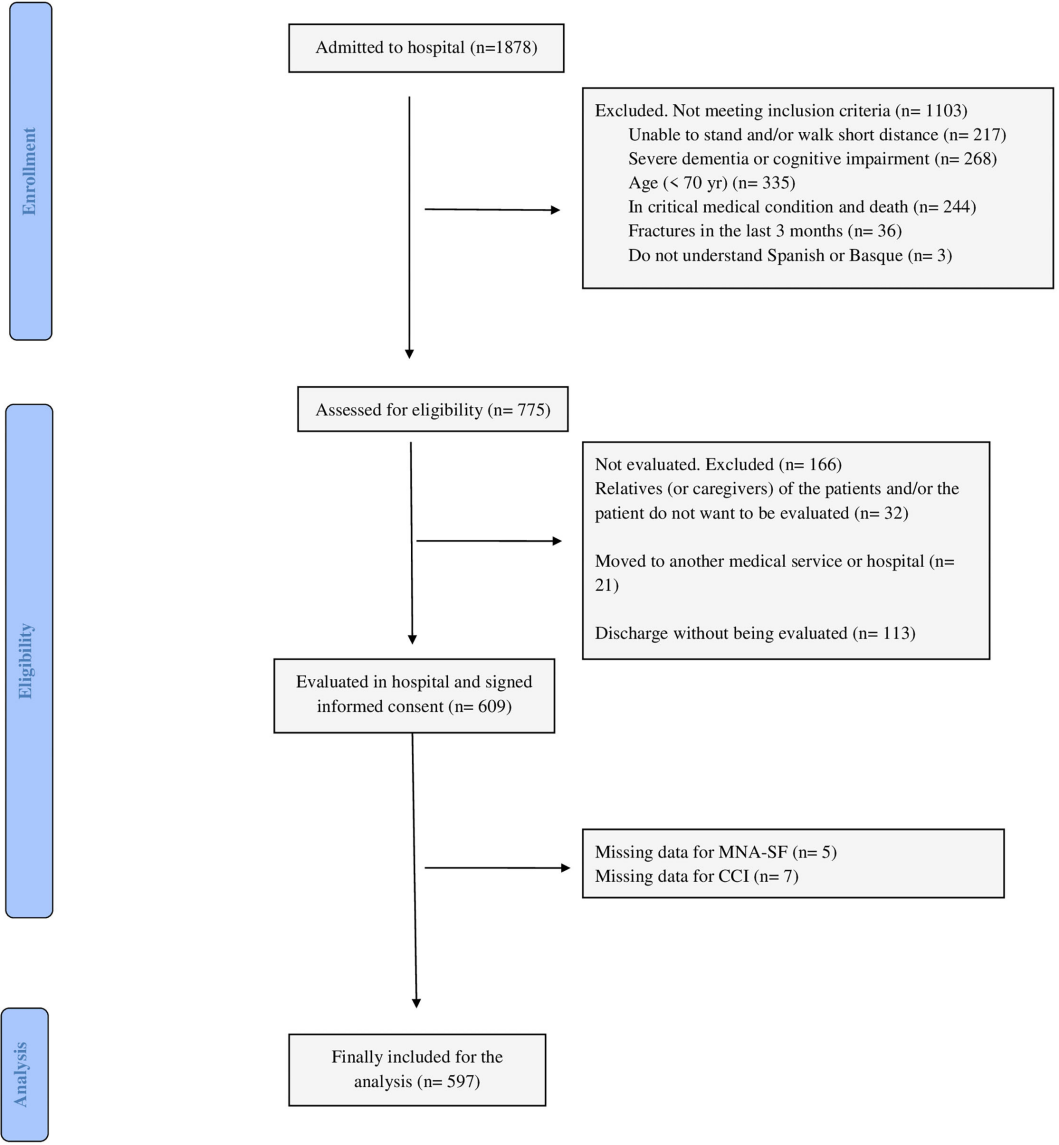
Flow diagram of participants.

Throughout the study duration 1,878 patients were admitted to the internal medicine service, out of which 1,103 (58.7%) inpatients did not meet the inclusion criteria, whereas 775 (41.3%) were eligible for evaluation, and, finally, 597 patients (98.0% of the eligible patients) were finally included in the analyses. The flowchart of the study and the reasons for the participant exclusion are detailed in Figure 1.

### Data Collection

Patients' clinical records were revisited to assess their medical history and the number of drugs given to the patients upon admission to the hospital. Polypharmacy was considered as the routine use of ≥5 drugs (31).

### Comorbidity Risk

Comorbidity burden was defined according to the CCI (32). The estimation of this index was based on age (divided into 5 ranges) and 17 different categories of comorbidity (32). Each age range and category had an associated score (from 1 to 6, the latter based on the severity of the condition), and then all were summed, contributing to the total score (32). Thereafter, 3 different categories were defined to classify comorbidity risk within patients: (1) 1–2 points mild risk, (2) 3–4 points moderate risk, and (3) ≥5 points severe risk (32). In the current study, all the participants were ≥70 years old, thereby we did not have any patients scoring 1–2 points due to the age-adjusted scoring (32).

### Nutritional Assessment

The MNA-SF questionnaire was used to assess patients' nutritional status by directly asking the patients and/or their caregivers. The MNA-SF is widely used in clinical settings and it has shown a high sensitivity (33, 34). For the current study, those at risk of malnutrition and who are malnourished were grouped together into “malnutrition or risk of malnutrition” category, as both are considered risk factors within the older adult population, and the remaining category was “normal nutritional status.”

### Physical Function Assessment

Two different tests were used to assess physical function: handgrip strength and the SPPB. Dominant handgrip strength (kg) was measured by a handheld dynamometer (JAMAR® PLUS + Hand dynamometer) in a seating position, as has been proposed for older adults in clinical practice (35). Those patients whose handgrip strength was ≤ P25 as compared with reference percentile values (36) were classified as unfit.

The SPPB clinical tool was chosen to measure physical function (37). The SPPB consists of 3 subtests: (1) the standing balance test, (2) the gait speed test, and (3) the 5 times sit-to-stand test. The total SPPB score ranges from 0 to 12, with higher scores reflecting better functional status, and it is divided into 4 categories: from 0 to 3, from 4 to 6, from 7 to 9, and from 10 to 12 points (37). It has been proposed that scores ≤ 9 points might help to detect frail older adults (38, 39). Hence, it has also been shown that scores below 10 points are associated with an increased risk of death (25). Thus, for the current study, it was decided to classify scores ranging from 0 to 9 as “frail” and scores ranging from 10 to 12 as “non-frail” (25, 38, 39).

### Statistical Analysis

The Kolmogorov–Smirnov test was employed to verify the distribution of the variables, and those with non-normal distribution were logarithmically transformed [i.e., age, body mass (kg), MNA-SF score, handgrip strength (kg), and SPPB total score]. Differences in socio-demographic and clinical characteristics between patients at moderate and severe comorbidity risk were analyzed using the independent Student *t-*test and the chi-square test for continuous and categorical variables, respectively.

A linear regression analysis was used to examine the association between the dependent (CCI) and independent (performance in fitness tests and MNA-SF score) variables and data were tested for gender interaction. An analysis of variance (polynomial) was done to examine the synergetic association of nutritional status and performance within each physical test with CCI by Bonferroni adjustment. Binary logistic regression models were carried out to analyze the likelihood of being at severe comorbidity risk according to the physical condition (unfit or frail vs. fit or non-frail) as well as the nutritional status (at risk of malnutrition or who were malnourished vs. normal nutritional status).

All statistical analyses were done using the statistical software SPSS version 20.0 (SPSS Inc., Chicago, IL, USA) with a level of significance of α = 0.05. Data are expressed as means ± SEM.

## Results

Table 1 shows the characteristics of participants by comorbidity risk categories. Briefly, those patients with severe risk of comorbidity were significantly older and had significantly higher rates of polypharmacy than those with moderate risk (all *p* < 0.001, Table 1).

**Table 1:** Characteristics of participants in the study by comorbidity risk according to the Charlson Comorbidity Index (CCI).

	***N***	**Whole sample**	***N***	**Moderate risk of comorbidity**	***N***	**Severe risk of comorbidity**	***P***
Age (years)	597	84.3 (6.8)	103	78.3 (5.8)	494	85.5 (6.3)	<0.001^†^
Female (*N*, %)	597	300, 50.3	103	59, 57.3	494	241, 48.8	0.117
Body mass (kg)^a^	583	67.2 (13.3)	100	69.5 (12.9)	483	66.7 (13.3)	0.050^†^
Number of drugs	597	7.2 (3.7)	103	5.1 (3.5)	494	7.7 (3.6)	<0.001^†^
Polypharmacy (*N*, %)	597	448, 75.0	103	51, 49.5	494	397, 80.4	<0.001
Depression (*N*, %)	597	55, 9.2	103	14, 13.6	494	41, 8.3	0.145
Diseases							
Hypertension (*N*, %)	597	441, 73.9	103	64, 62.1	494	377, 76.3	<0.05
CVD (*N*, %)	597	177, 29.6	103	4, 3.9	494	173, 35.0	<0.001
COPD (*N*, %)	597	120, 20.1	103	5, 4.9	494	115, 23.3	<0.001
Diabetes (*N*, %)	597	203, 34.0	103	12, 11.7	494	191, 38.7	<0.001
Kidney disease (*N*, %)	597	107, 17.9	103	2, 1.9	494	105, 21.3	<0.001
Hepatic disease (*N*, %)	597	13, 2.2	103	1, 1.0	494	12, 2.4	0.223
Neoplasia (*N*, %)	597	122, 20.4	103	0, 0.0	494	122, 24.7	<0.001
Dementia (*N*, %)	597	23, 3.9	103	2, 1.9	494	21, 4.3	0.161
Parkinson (*N*, %)	597	19, 3.2	103	6, 5.8	494	13, 2.6	0.191
*Nutritional Status*							
MNA-SF score	597	10.0 (2.5)	103	10.9 (2.1)	494	9.8 (2.6)	<0.001^†^
Normal nutritional status (*N*, %)	597	205, 34.3	103	51, 49.5	494	154, 31.2	<0.001
At risk of malnutrition (*N*, %)	597	288, 48.2	103	44, 42.7	494	244, 49.4	
Malnourished (*N*, %)	597	104, 17.4	103	8, 7.8	494	96, 19.4	
*Physical Function*							
Handgrip (kg)^b^	596	19.5 (8.2)	103	22.2 (8.8)	493	19.0 (8.0)	<0.005^†^
SPPB total score ^c^	591	5.4 (3.1)	102	7.2 (2.9)	489	5.0 (3.0)	<0.001^†^
SPPB categorized							
0–3 (*N*, %)	591	188, 31.8	102	13, 12.7	489	175, 35.8	<0.001
4–6 (*N*, %)	591	191, 32.3	102	29, 28.4	489	162, 33.1	
7–9 (*N*, %)	591	135, 22.8	102	30, 29.4	489	105, 21.5	
10–12 (*N*, %)	591	77, 13.0	102	30, 29.4	489	47, 9.6	

*Values are means and standard deviations unless otherwise indicated*.

*CVD, cardiovascular disease; COPD, chronic obstructive pulmonary disease; MNA-SF score, Mini Nutritional Assessment-Short Form score; SPPB total score, Short Physical Performance Battery total score*.

*p refers to differences between patients at moderate and severe comorbidity risk analyzed by the t-test for independent samples in continuous variables and Chi-squared test for categorical variables.

*†Means and standard deviations are presented for not transformed variables to ease interpretation, but p-values were obtained by the t-test for independent samples with logarithmically transformed continuous variables*.

*^a^Data were missing for 14 patients*.

*^b^Data was missing for 1 patient*.

*^c^Data were missing for 6 patients*.

Participants with severe risk of comorbidity scored significantly lower in the MNA-SF (*p* < 0.001) and performed significantly worse within handgrip and SPPB tests (all *p* < 0.005) than those patients with moderate comorbidity risk.

### Association of Comorbidity Risk With Nutritional Status and Physical Function

Table 2 shows the associations of CCI scores with the nutritional status and performance-based physical tests. Higher punctuation in the MNA-SF (*p* < 0.05) as well as better performance within handgrip strength (*p* < 0.005) and the SPPB (*p* < 0.005) were significantly associated with lower CCI scores among men and women.

**Table 2:** Linear regression of comorbidity risk assessed by the CCI with nutritional status and physical function by sex.

		**Nutritional status**			**Physical function**					
		**MNA-SF (total score)**			**Handgrip (kg)**			**SPPB (total score)**		
		**β**	***Error and R^**2**^***	***P***	**β**	***Error and R^**2**^***	***P***	**β**	***Error and R^**2**^***	***P***
CCI (lineal)	Males	−0.125 IC 95% (−0.281 to −0.013)	0.068/0.016	<0.05	−0.185 IC 95% (−0.286 to −0.070)	0.055/0.034	<0.005	−0.200 IC 95% (−0.151 to −0.042)	0.028/0.040	<0.005
	Females	−0.154 IC 95% (−0.231 to −0.036)	0.050/0.024	<0.05	−0.265 IC 95% (−0.266 to −0.110)	0.040/0.070	<0.001	−0.306 IC 95% (−0.175 to −0.082)	0.024/0.094	<0.001

*CCI, Charlson comorbidity index; MNA-SF (total score), Mini Nutritional Assessment-Short Form (total score); SPPB (total score), Short Physical Performance Battery (total score)*.

*Unadjusted linear regression tests*.

*Linear regressions were calculated with the logarithmically transformed variables*.

### Likelihood for Being at Severe Risk of Comorbidity According to Nutritional Status and Physical Function

**Figure 2:**
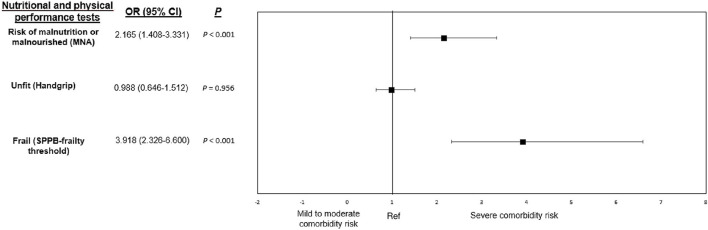
Odd ratios and 95% confidence intervals (95% *CI*) for being classified as at severe comorbidity risk [CCI ≥ 5, (32)] according to the nutritional status (MNA-SF) and different performance-based physical tests (handgrip and SPPB). MNA-status, Mini Nutritional Assessment-status; SPPB-frailty threshold, Short Physical Performance Battery-frailty threshold. Unadjusted odds ratios. Nutritional assessment by the Mini Nutritional Assessment-Short Form questionnaire (scores ≤ 11 at risk of malnutrition or malnourished); handgrip unfit assessment according to Dodds et al. (36) percentiles (≤ P25 unfit); frailty assessment according to the Short Physical Performance Battery frailty threshold (scores ≤ 9 frail). Ref: scores > 11 normal nutritional status; >P25 fit; scores > 9 non-frail.

Figure 2 shows the likelihood of being at severe risk of comorbidity according to the nutritional status and the physical function. It was observed that the likelihood of being at severe risk of comorbidity was 2.0 times higher in patients at risk of malnutrition or those who were malnourished (*OR*: 2.165, 95% *CI*: 1.408–3.331, *p* < 0.001) and 3.5 times higher in frail patients (*OR*: 3.918, 95% *CI*: 2.326–6.600, *p* < 0.001). In contrast, being unfit for handgrip strength did not increase the risk of being at severe risk of comorbidity (*OR*: 0.988, 95% *CI*: 0.646–1.512, *p* = 0.956).

### Comorbidity Risk According to the Combination of the Nutritional Status and Physical Function of Participants

**Figure 3:**
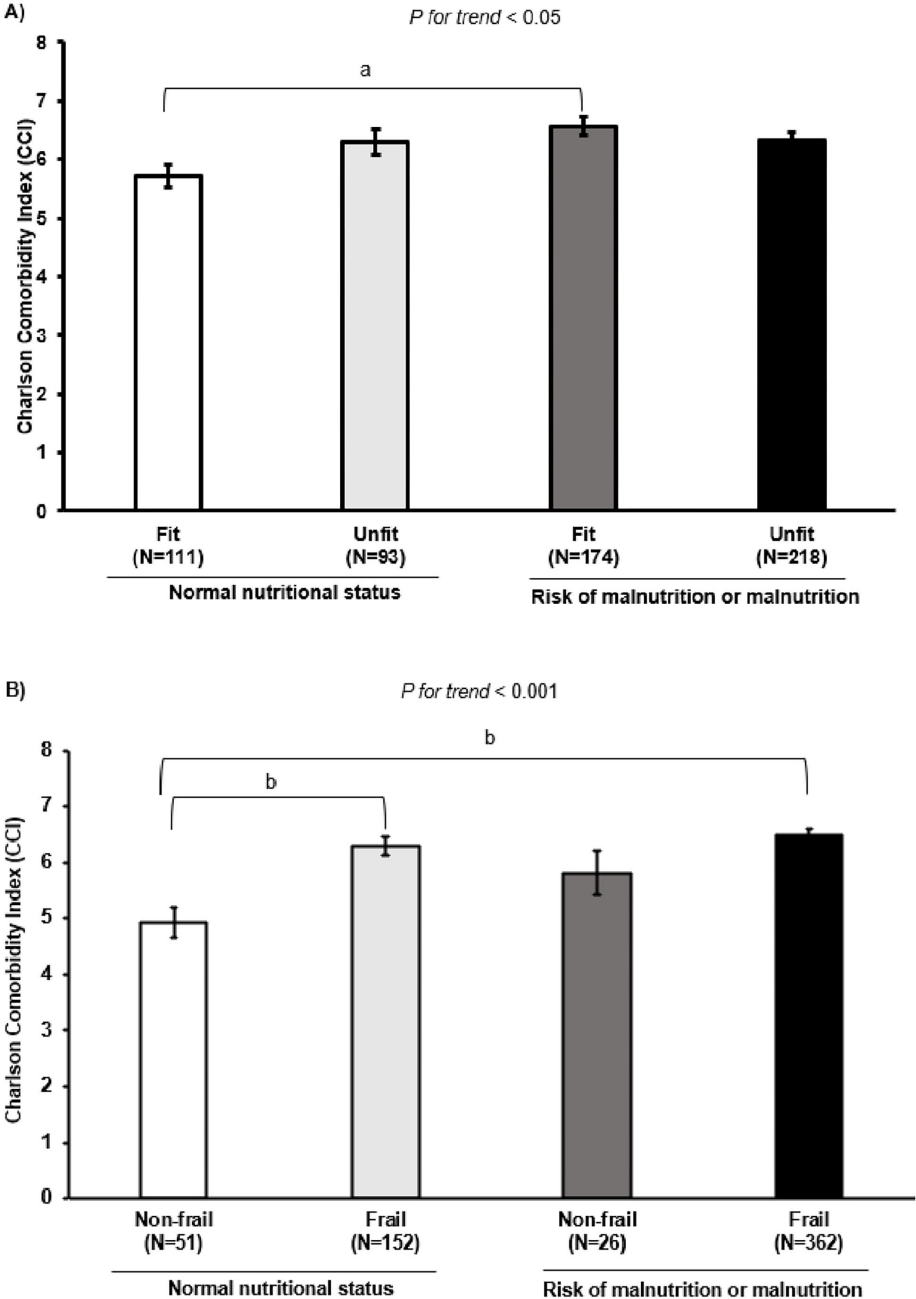
Differences among comorbidity risk according to the nutritional status combined with several physical parameters. **(A)** Nutritional status combined with fitness categories assessed by handgrip strength (kg) [ ≤ P25 unfit, (36)]. **(B)** Nutritional status combined with frailty categories (SPPB score ≤ 9 frail). Unadjusted analysis of variance (polynomial). ^a^*p* < 0.005; ^b^*p* < 0.001.

Combined associations of nutritional status and performance-based categories on comorbidity risk are shown in Figure 3. It was observed that patients at risk of malnutrition or who were malnourished had higher CCI scores regardless of being fit or unfit according to handgrip strength (*p* for trend < 0.05, panel A). Among fit patients, those with normal nutritional status had lower CCI scores than those at risk of malnutrition or who were malnourished (5.7 vs. 6.6, respectively, *p* < 0.005, panel A). Regarding the SPPB frailty threshold, patients classified as frail had higher CCI despite their nutritional status (*p* for trend < 0.001, panel B). Among those participants with normal nutrition status, those classified as frail had higher CCI scores than non-frail patients (6.3 vs. 4.9, respectively, *p* < 0.001, panel B). Nevertheless, those frail patients at risk of malnutrition or who were malnourished also had higher CCI scores in comparison to their no-frail counterparts with normal nutritional status (6.5 vs. 4.9, respectively, *p* < 0.001, panel B).

## Discussion

The primary findings of the current study were that those hospitalized older adults aged ≥70 at risk of malnutrition or who were malnourished and frail according to the SPPB frailty threshold, had a significantly higher risk of being at severe risk of comorbidity than their peers with normal nutritional status and who were not frail (SPPB frailty threshold). Hence, older inpatients (≥70 years old) classified as non-frail had lower values of CCI regardless of their nutritional status. Thus, nutritional status and physical function assessment might help to predict indirectly mortality risk among the older adult population in clinical settings.

Aging is considered an important risk factor for most diseases and conditions limiting healthspan (40). Having used the CCI that includes an age-associated score according to age ranges limits the comparison with other studies (28–30) and makes it difficult to account for the contribution of any disease to the CCI score within each inpatient. So, to observe the pattern of chronic diseases within the older inpatients in this study (≥70 years old), age was removed from the CCI scoring. Similarly, 87.6% of the inpatients in this study showed at least one chronic disease, and from those, 74.8% scored ≥2 for the CCI showing more than one chronic disease and/or a severe chronic disease. Of 74.8% of the older inpatients, 81.3% were ≥80 years old. These results are in line with a previous study conducted in Italy concluding that 86% of the adults older than 65 years lived with at least one chronic disease (2), and with other studies showing that multimorbidity increases with age (1, 3). Thus, these results reinforce aging as an important risk factor for increasing the comorbidity burden. Hence, in the current study, patients at severe risk of comorbidity were significantly older, had higher rates of polypharmacy, and showed higher rates of chronic diseases as well as worse nutritional status and physical function compared to those at moderate risk of comorbidity.

Our results show that higher punctuation in the MNA-SF and performance-based physical tests were inversely associated with the CCI score among older inpatients. Previous studies aiming to measure the health-related consequences of malnutrition used different data other than the CCI, such as the length of hospital stay (41), readmission rates (12), the morbidity of a specific disease (8), or short- (11, 14) and long-term mortality (15). There are only few studies using the CCI as a primary variable for that aim, and in contrast to our findings, they reported no association between nutritional status and comorbidity (29, 30). The different methodology used to assess nutritional assessment in those studies (29, 30) limits comparison with the results obtained in this study. Nevertheless, as previously reported, the MNA-SF test performed well in predicting unfavorable clinical outcomes (42) and is proposed to be the first choice for geriatric hospital patients (43). Hence, this might be reflected by the 2-fold increase in severe comorbidity risk seen in malnourished older adults in the current study.

Similarly, handgrip strength has been proposed as a health biomarker (18). Hence, the previous studies showed an inverse association between handgrip strength and multimorbidity (19, 20), which is in line with the findings of the current study, as every increase in handgrip strength was negatively associated with CCI score. However, the current study failed to show an increased risk for severe comorbidity according to handgrip strength. This might be due to the handgrip percentiles used as a reference, which were based on normative data (36), and/or due to the sex-interaction seen for handgrip strength, as the inverse association between handgrip strength and the CCI was stronger among older women in the current study. Similar findings were observed in the study of Volaklis et al. (19), where low handgrip strength was associated with an increased odds of multimorbidity among older women, but not men. Physiological mechanisms were suggested to explain sex-related differences in the relationship between handgrip strength and morbidity (19). Nevertheless, although an increased risk for mortality was shown along with a decline in handgrip strength among older adults (21, 22), it has also been suggested that the relation between muscle strength and mortality is not direct (21) and that, behind that interaction, there might be other factors underlying mortality (21), such as the number of diseases and/or their severity, which is accounted within the CCI scoring. Thus, this hypothesis might be reinforced by this study, although further research is needed to confirm it.

The SPPB has gained attention due to its ability to predict mortality risk (25, 26) and its association with frailty (38). Frailty has been linked to multimorbidity in several studies as shown by a recent systematic review and meta-analysis (44), but none of those studies used the SPPB to assess frailty. Although this limits comparison, it seems that the current study is in line with those results. Hence, being frail according to the SPPB increased the risk for severe comorbidity by almost 4-fold to that seen in non-frail hospitalized older adults in this study. Veronese et al. (26) showed that a low SPPB score predicted mortality. Thus, considering that the CCI is often used to predict mortality, results from the current study might be in agreement with that stated by Veronese et al. (26).

Lastly, an interesting finding from the current study is the synergetic effect observed between the nutritional status and performance-based physical tests. Indeed, according to handgrip strength and nutritional status, hospitalized older adults at risk of malnutrition or malnourished ones had higher CCI scores regardless of being fit or unfit according to handgrip strength. To our knowledge, there is only one study carried out in a care home for veterans reporting a synergistic effect of malnutrition and low handgrip strength on 4-year all-cause mortality (23). The authors reported that malnourished individuals with low handgrip strength were at 3.14 times higher risk of mortality (23), and that malnutrition was an independent risk factor for 4-year all-cause mortality (23). In our study, in contrast, frail patients showed higher CCI scores despite being well-nourished or malnourished. To our knowledge, there is no previous study combining nutritional status and frailty status, according to the SPPB threshold, of hospitalized older adults to compare with. Nevertheless, the results of the current study support frailty as a state of high vulnerability (45) and, thereby, these inpatients aged ≥70 years might benefit from an exercise training program after hospitalization (46, 47). There is one recent study carried out on older adults admitted to hospital for the acute coronary syndrome, where they analyzed the incremental value from adding the MNA-SF as well as the SPPB to the model for predicting all-cause mortality (48). The MNA-SF significantly improved the ability of the model to predict all-cause mortality, but the discrimination ability significantly improved with the addition of MNA-SF to the model with SPPB (48). This needs to be further studied as the study was based on older adults with a specific characteristic (acute coronary syndrome), but it arises new insights into the use of the MNA-SF and the SPPB in clinical settings for predicting adverse clinical outcomes, and it reinforces our results.

To the best of our knowledge, this is the first study carried out on hospitalized older adults (≥70 years old) examining the association between their nutritional status and physical function with the CCI score. Hence, nutritional status and physical function assessments were conducted by using the most widely easy-to-use tools recommended for geriatric hospitalized adults, the MNA-SF and handgrip strength and the SPPB, respectively. Another strength of the current study could be considered the large sample size (*N* = 597) as required for studies examining the prognosis of comorbidity (6). However, some limitations should be recognized. First, the cross-sectional design of the study limits the determination of any causality. Second, although the CCI is widely used in clinical settings, it was developed in a specific population different from the sample of the current study (32), and scores are often obtained from medical records that, although being more complete than other sources, might have added some bias by recording some diseases (6). Third, the reference percentiles for handgrip strength that were used might have limited the results (36). Thus, future studies regarding handgrip strength and comorbidity risk will be required to contrast the results of the current study. Finally, this study cannot be extrapolated to other older adult populations not meeting the inclusion criteria for this study and from other clinical settings or to community-dwelling older adults. So, future studies in populations with different clinical characteristics should be conducted.

## Conclusions

The current study confirms that malnutrition and poor physical function are associated with increased comorbidity (CCI) in hospitalized older adults aged 70 and older. Hence, being malnourished or frail increased the risk to be classified as a severe comorbidity (CCI). Both, along with other syndromes, are widespread conditions in older adults and are often overlapped (49). This hinders the identification of risk factors contributing to comorbidity and, finally mortality, within the older adult population. However, the results in this study suggest that frailty, according to the SPPB frailty threshold, might be a major contributor to the CCI increase than the nutritional status in hospitalized older adults. Nevertheless, the current study reinforces the use of the MNA-SF and the SPPB in geriatric hospital patients as they might help to predict poor clinical outcomes and thus indirectly predict post-discharge mortality risk (50). Thereby, including both tests in the routine clinical practice will help to better screen those patients at risk and will also permit to better monitor their evolution during and after hospitalization.

## Data Availability Statement

The original contributions presented in the study are included in the article, further inquiries can be directed to the corresponding authors.

## Ethics Statement

The studies involving human participants were reviewed and approved by the Clinical Research Ethics Committee of the Araba University Hospital (CEIC-HUA: 2017-021). The patients/participants provided their written informed consent to participate in this study.

## Author Contributions

IL and B-BA designed the study. MA, IE, and MU collected the data. MA, MM, IL, and B-BA interpreted the data and drafted the manuscript. MA, MM, IE, MU, AR-L, AD, IL, and B-BA have approved the submitted version and agree to be personally accountable for the author's own contributions and for ensuring that questions related to the accuracy or integrity of any part of the work, even ones in which the author was not personally involved, are appropriately investigated, resolved, and documented in the literature. All authors contributed to the article and approved the submitted version.

## Funding

This study was supported by the Basque Government (2016111138). MA was supported by a grant from the University of the Basque Country (PIF17/186) and IE was supported by a grant from the University of the Basque Country in collaboration with the University of Bordeaux (UBX) (PIFBUR16/07).

## Conflict of Interest

The authors declare that the research was conducted in the absence of any commercial or financial relationships that could be construed as a potential conflict of interest.

## Publisher's Note

All claims expressed in this article are solely those of the authors and do not necessarily represent those of their affiliated organizations, or those of the publisher, the editors and the reviewers. Any product that may be evaluated in this article, or claim that may be made by its manufacturer, is not guaranteed or endorsed by the publisher.

## Abbreviations

CCI: Charlson Comorbidity Index
MNA-SF: Mini Nutritional Assessment-Short Form
SPPB: short physical performance battery
CVD: cardiovascular disease
COPD: chronic obstructive pulmonary disease.
